# Lack of ZnT8 protects pancreatic islets from hypoxia- and cytokine-induced cell death

**DOI:** 10.1530/JOE-21-0271

**Published:** 2022-01-11

**Authors:** Maria Karsai, Richard A Zuellig, Roger Lehmann, Federica Cuozzo, Daniela Nasteska, Edlira Luca, Constanze Hantel, David J Hodson, Giatgen A Spinas, Guy A Rutter, Philipp A Gerber

**Affiliations:** 1 Department of Endocrinology, Diabetology and Clinical Nutrition, University Hospital Zurich (USZ) and University of Zurich (UZH), Zurich, Switzerland; 2 Institute of Metabolism and Systems Research (IMSR) and Centre of Membrane Proteins and Receptors (COMPARE), University of Birmingham, Birmingham, UK; 3 Centre for Endocrinology, Diabetes and Metabolism, Birmingham Health Partners, Birmingham, UK; 4 Medizinische Klinik und Poliklinik III, University Hospital Carl Gustav Carus Dresden, Dresden, Germany; 5 Section of Cell Biology and Functional Genomics, Division of Diabetes, Endocrinology and Metabolism, Department of Metabolism, Digestion and Reproduction, Imperial College London, London, UK; 6 CR-CHUM, University of Montreal, Montreal, QC, Canada; 7 Lee Kong Chian School of Medicine, Nanyang Technological University, Singapore, Singapore

**Keywords:** beta cell, hypoxia, inflammation, pancreatic islet, zinc, zinc transporter

## Abstract

Pancreatic β-cells depend on the well-balanced regulation of cytosolic zinc concentrations, providing sufficient zinc ions for the processing and storage of insulin, but avoiding toxic effects. The zinc transporter ZnT8, encoded by *SLC30A8,*is a key player regarding islet cell zinc homeostasis, and polymorphisms in this gene are associated with altered type 2 diabetes susceptibility in man. The objective of this study was to investigate the role of ZnT8 and zinc in situations of cellular stress as hypoxia or inflammation. Isolated islets of WT and global ZnT8^−/−^ mice were exposed to hypoxia or cytokines and cell death was measured. To explore the role of changing intracellular Zn^2+^ concentrations, WT islets were exposed to different zinc concentrations using zinc chloride or the zinc chelator *N*,*N*,*N′*,*N′*-tetrakis(2-pyridinylmethyl)-1,2-ethanediamine (TPEN). Hypoxia or cytokine (TNF-α, IFN-γ, IL1-β) treatment induced islet cell death, but to a lesser extent in islets from ZnT8^−/−^ mice, which were shown to have a reduced zinc content. Similarly, chelation of zinc with TPEN reduced cell death in WT islets treated with hypoxia or cytokines, whereas increased zinc concentrations aggravated the effects of these stressors. This study demonstrates a reduced rate of cell death in islets from ZnT8^−/−^ mice as compared to WT islets when exposed to two distinct cellular stressors, hypoxia or cytotoxic cytokines. This protection from cell death is, in part, mediated by a reduced zinc content in islet cells of ZnT8^−/−^ mice. These findings may be relevant for altered diabetes burden in carriers of risk *SLC30A8* alleles in man.

## Introduction

Pancreatic β-cells are highly dependent on the micronutrient zinc for the processing and storage of insulin (Chabosseau & Rutter 2016), which is then released in response to rising glucose concentrations. The cytosolic concentration of free Zn^2+^ in pancreatic β-cells is 400–500 pM (Bellomo *et al.* 2011) and is much higher in insulin-secreting granules, reaching high micromolar (Vinkenborg *et al.* 2009) to the millimolar range (Foster *et al.* 1993).

High concentrations of intracellular zinc, resulting from exogenous administration or release from intracellular stores, are toxic and potentially lethal for cells (Plum *et al.* 2010). This has been shown not only in different tissues, such as cortical neurons (Kim *et al.* 1999), but also in pancreatic islet cells, where zinc is cytotoxic in a dose-dependent manner (Kim *et al.* 2000).

To reach intracellular concentrations sufficient for proper function, but at the same time avoiding the toxic effects of zinc, intracellular zinc and its flux from/into different organelles is tightly regulated by zinc-binding and -transporting proteins. Three main protein families are responsible for the maintenance of zinc homeostasis: the metallothioneins (MTs), the zinc importers (ZIP, SLC39A) and the zinc transporters (ZnT, SLC30A) (Chabosseau & Rutter 2016). Zinc transporter 8 (ZnT8), encoded by the *SLC30A8/Slc30a8* gene, is of particular interest in terms of islet cell function (Rutter & Chimienti 2015). In β-cells, ZnT8 is the most abundantly expressed zinc transporter and is responsible for the efflux of Zn^2+^ from the cytosol. ZnT8 transporters are mainly located on the membrane of insulin-secreting granules (Lichten & Cousins 2009). Furthermore, the expression of ZnT8 in high levels is quite specific for pancreatic islet cells (α- and β-cells (Chimienti *et al.* 2004, Solomou *et al.* 2015, Ghazvini Zadeh *et al.* 2020)).

In 2007, a genome-wide association study identified a non-synonymous polymorphism in the zinc transporter *SLC30A8* gene as being linked with an increased risk of type 2 diabetes mellitus (T2DM) (Sladek *et al.* 2007). Subsequently, it was shown that global and β-cell specific *Slc30a8* knockout mice display age-, sex- and diet-dependent abnormalities of various degree in glucose tolerance, insulin secretion and body weight (Nicolson *et al.* 2009, Wijesekara *et al.* 2010). Later studies, however, provided evidence for a protective role of loss-of-function mutations in *SLC30A8* with regard to T2DM risk (Flannick *et al.* 2014, Dwivedi *et al.* 2019).

We have shown previously that islets exposed to hypoxia exhibited a decreased expression of *Slc30a8* and lowered zinc concentrations, while cell survival was enhanced in islets of global *Slc30a8* knockout mice (Gerber *et al.* 2014). This was surprising since pancreatic islets are highly dependent on oxidative metabolism (resulting from glycolytic flux) for ATP synthesis, and oxygen deprivation has a major impact on their function and survival (Sekine *et al.* 1994). The question remained as to why changes in zinc transporter abundance are potent enough to alter this cellular response to hypoxia. Thus, with the present study, we sought to explore the mechanism of enhanced islet cell survival in islets of *Slc30a8* knockout mice under hypoxic conditions or other situations of islet cell stress.

## Materials and methods

### Animals

Female CD1 mice were purchased from Charles River. Global female ZnT8^−/−^ mice on a mixed 129Sv/C57BL/6J background have previously been described (Nicolson *et al.* 2009). Mice were euthanized at the specified age by cervical dislocation. Female WT littermates were used as a control to ZnT8^−/−^ mice. Animal experiments were conducted following the EU Directive for animal experiments. All animal procedures were approved by the Cantonal Veterinary office in Zurich, Switzerland (164/2018), or the Animals (Scientific Procedures) Act 1986 of the United Kingdom (Personal Project Licences P2ABC3A83 and PP1778740), as well as the University of Birmingham’s Animal Welfare and Ethical Review Body (AWERB).

### Islets isolation and culture

Mouse islets were prepared as in Ravier and Rutter (2005). Briefly, pancreata were perfused via the pancreatic duct with a NB8 collagenase solution (SERVA, Uetersen, Germany). Pancreata were digested at 37°C for 10 min and separated using a histopaque gradient (Sigma-Aldrich). Islets were handpicked under a microscope and cultured overnight in RPMI 1640 containing 11.1 mM glucose.

### Experiments in isolated islets

Isolated pancreatic islets were exposed to hypoxia (1% O_2_, 5% CO_2_ and 94% N_2_) or normoxia (21% O_2_, 5% CO_2_ and 74% N_2_) using a tissue culture incubator (CB-60, Binder). Zinc deprivation was achieved using 50 μM N,N,N′,N′-tetrakis(2-pyridylmethyl)ethylenediamine (TPEN, Sigma-Aldrich). Zinc supplementation was performed using 600 μM zinc chloride solution (ZnCl_2_, Sigma-Aldrich). Cytokine treatment was performed using a mixture of cytokines: 1000 U/mL Tumour necrosis factor-α (TNF-α; ThermoFischer), 1000 U/mL Interferon-γ (IFN-γ; ThermoFischer) and 50 U/mL interleukin-1β (IL-1β; ThermoFischer) (Jain *et al.* 2015). All treatments were conducted for 24 h.

### Live/Dead cell assay

Following treatment, islets were incubated for 15 min in PBS containing 3 μM calcein-AM and 2.5 μM propidium iodide (PI) at 37°C (04511 Cellstain double staining kit, Sigma-Aldrich), before detection of absorbance/emission at 491/525 and 561/620 nm, respectively (Gerber *et al.* 2014). ImageJ was used for data capture and analysis, where the islet area occupied by dead cells (PI) was calculated and expressed as a unitary ratio vs that occupied by all (live and dead) cells (PI plus calcein-AM).

### Assessment of apoptosis and cell proliferation

Isolated islets were cultured for 24 h in RPMI 1640 (+10% FBS + 1% P/S) medium containing 50 μM TPEN, or 600 μM ZnCl_2_, or a cytokine mixture containing 1000 U/mL TNF-α, 50 U/mL interleukin-1β and 1000 U/mL interferon-γ or a combination of these.

After being fixed in 4% paraformaldehyde, the islets were subjected to antigen retrieval using citrate buffer. Subsequently, TUNEL staining was performed using the DeadEnd Fluorometric TUNEL System (Promega), according to the manufacturer’s instructions and co-staining for PCNA was carried out using mouse anti-proliferating cell nuclear antigen (PCNA) 1:500 (Cell Signaling Technology) as primary antibody and anti-mouse Alexa Fluor 568 1:500 (Thermo Fisher Scientific) as secondary antibody. The islets were mounted on SUPERFROST slides using VECTASHIELD HardSet with DAPI (Vector Laboratories, Burlingame, USA).

Images were acquired using a Zeiss LSM780 meta-confocal microscope using PMT spectral detectors and a 25×/0.8 immersion Plan-Apochromat objective. Excitation was delivered at λ = 405 nm, λ = 488 nm and λ = 561 nm for DAPI, TUNEL + staining (fluorescein-12-dUTP) and Alexa Fluor 568, respectively. Emitted signals were detected at λ = 415–533 nm, λ = 488–566 nm and λ = 571–648 nm for DAPI, TUNEL and Alexa Fluor 568, respectively. Islets were size-matched for analysis, and changes in apoptosis and proliferation normalized against controls as fold change.

### Imaging of cytosolic Zn^2+^ concentrations with FluoZin-3AM

Islets were incubated for 1 h in 6 μM of the cell-permeable dye FluoZin-3AM (Invitrogen) dissolved in DMSO (0.01%, w/v), buffered by bicarbonate buffer containing 11 mM glucose. Imaging was performed with a ZEISS microscope, FluoZin-3AM was excited at 470 nm. ImageJ (https://imagej.net/Fiji, October 2016) was used for data capture and analysis. Results were normalized to the background fluorescence.

### Measurements of glycated haemoglobin (HbA1c)

Mouse tail blood was collected and HbA1c was measured on a DCA 2000 Plus biochemical analyzer (Bayer) (Zehetner *et al.* 2008).

### Glucose-stimulated insulin secretion (GSIS) from isolated islets

Isolated islets were cultured overnight in normal culture medium (RPMI 1640 containing 11.1 mM glucose, 100 U/mL penicillin, 100 µg/mL streptomycin, 2 mM l-alanyl-l-glutamine and 10% FBS). Islets were pre-incubated for 30 min in Krebs-Ringer Hepes bicarbonate (KRHB) buffer (131 mM NaCl, 4.8 mM KCl, 1.3 mM CaCl_2_•2H_2_O, 25 mM HEPES, 1.2 mM KH_2_PO_4_ and 1.2 mM MgSO_4_•7H_2_O with 0.5% BSA) with 3.3 mM glucose. Following that, GSIS was assessed by static incubation of islets in KRHB with 3.3 mM, followed by 16.7 mM and then again 3.3 mM glucose for 1 h at 37°C. After each incubation step, the medium was collected and subjected to ELISA (Mouse insulin ELISA, Mercodia, Sweden).

### Intraperitoneal glucose tolerance test (IPGTT)

Before the glucose tolerance test, mice were fasted overnight for 16 h. After determination of fasted blood glucose levels, each animal received an i.p. injection of saline glucose solution (2 g/kg body weight). The measurements were performed before and 15, 30, 45, 60, 90 and 120 min after glucose injection.

### RNA extraction and qPCR

Total RNA from ~50 islets was obtained using TRIzol reagent (Ambion by Life technologies) and reverse transcribed into cDNA using a high-capacity RNA-to-cDNA kit (Applied Biosystems by Thermo Fisher Scientific). cDNA was subject to qPCR using Power SYBR Green master mix (Applied Biosystems by Thermo Fisher Scientific) in a 7500 fast real-time PCR system (Applied Biosystems) and analysed by the comparative C_t_ method (primers are shown in Supplementary Table 1, see section on supplementary materials given at the end of this article). The expression of target genes was normalized to the expression of *Ppia* (encoding cyclophilin A).

### Cell line culture and Western blot analysis

EndoC-βH5 cells (Univercell Biosolutions, Toulouse, France) were handled according to manufacturer instructions. The cells were cultured for 1 week, following which they were treated with a mixture of TNF-α (10 ng/mL, ThermoFischer), IL1-β (50 U/mL, ThermoFischer) and IFN-γ (750 U/mL, ThermoFischer) for 24 h. Treated and untreated cells (*n* = 4) were lysed on ice with radioimmunoprecipitation assay (RIPA) buffer, sonicated for 15 s and then 40 µg of protein was loaded for immunoblotting and detected with antibodies against human ZnT8 (Proteintech, Manchester, UK) and γ-tubulin (Sigma-Aldrich).

### Data analysis

Data are presented as single data points and mean ± s.d., unless otherwise stated. The statistical significance of differences between groups was assessed by Student’s *t*-test/Mann–Whitney *U* test. Generalized multivariate analysis was used to assess the effect of different factors on islet cell death and in particular to separate the effect of islet size from other factors. ANOVA was used for comparisons between a higher number of groups. *P*-values < 0.05 were considered to be statistically significant. All statistical analyses were performed using Prism 8.04 software (GraphPad Software) and SPSS Statistics 26.0 software.

## Results

### Islet cells deficient of *Slc30a8* exhibit decreased cell death despite impaired long-term glucose control

**Figure 1:**
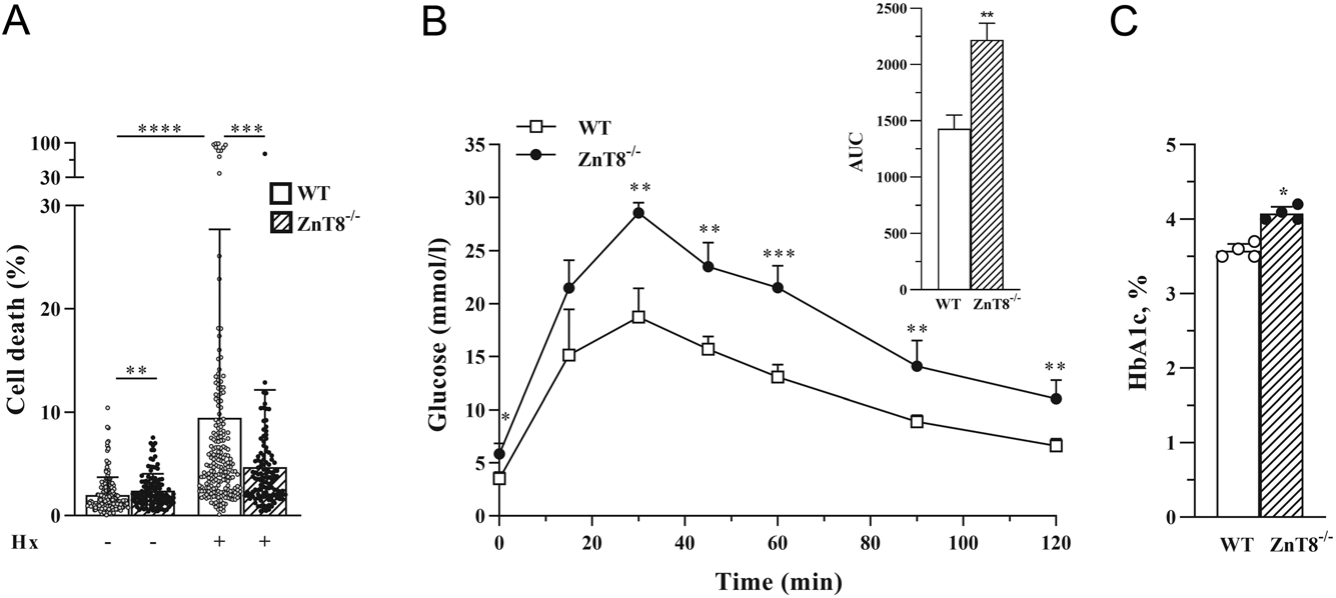
Loss of ZnT8 protects against hypoxia-mediated islet cell death despite impaired glucose control. (A). The proportion of dead cells in islets of WT and ZnT8^−/−^ mice at the age of ~25 weeks is depicted after incubation of islets for 24 h at normoxia (21% O_2_) or hypoxia (1% O_2_, Hx), *n* (islet number) ≥ 100 islets per condition. (B) A glucose tolerance test was performed after i.p. administration of glucose (2 g/kg body weight) in 48-week-old mice, *n*  = 4 per group. Corresponding areas under the curve (AUC) are shown. (C) Glucose control was assessed by HbA1c determination in WT and ZnT8^−/−^ mice at the age of ~25 weeks, *n* (number of mice) = 4 in each group. Data are expressed as mean ± s.d. ^*^P ≤ 0.05, ^**^P ≤ 0.01, ^***^P ≤ 0.001, ^****^P ≤ 0.0001. Generalized multivariate analysis (A), Student’s *t*-test (B) and Mann–Whitney *U* test (C) were performed.

We confirmed the previous results (Gerber *et al.* 2014) demonstrating that cell death was lowered in ZnT8*^−/−^* islets exposed to hypoxia (as a potent inducer of cell death) (Fig. 1A). In order to correct for a possible influence of islet size on cell death, generalized multivariate analysis was used.

Taking into consideration recent evidence for a protection against diabetes resulting out of a loss-of-function of ZnT8 (Dwivedi *et al.* 2019) and due to previously reported variations in dysglycaemia in mice deficient in ZnT8 (Rutter & Chimienti 2015, Rutter *et al.* 2016), we assessed short- and long-term glucose control in our mice deficient in ZnT8. It was hypothesized that a possible change of long-term glucose levels could lead to the change in the susceptibility of pancreatic islet cells to cell death described above. In accordance with some previously published results (Nicolson *et al.* 2009), the assessment of glucose tolerance as well as glycated haemoglobin revealed impaired glucose control and higher levels of HbA1c in ZnT8^−/−^ mice compared to WT mice (Fig. 1B and C), thus rejecting the hypothesis that reduced glucose levels underlie protection from cell death.

### Reduction of hypoxia-induced cell death in ZnT8-deficient islets depends on zinc

**Figure 2:**
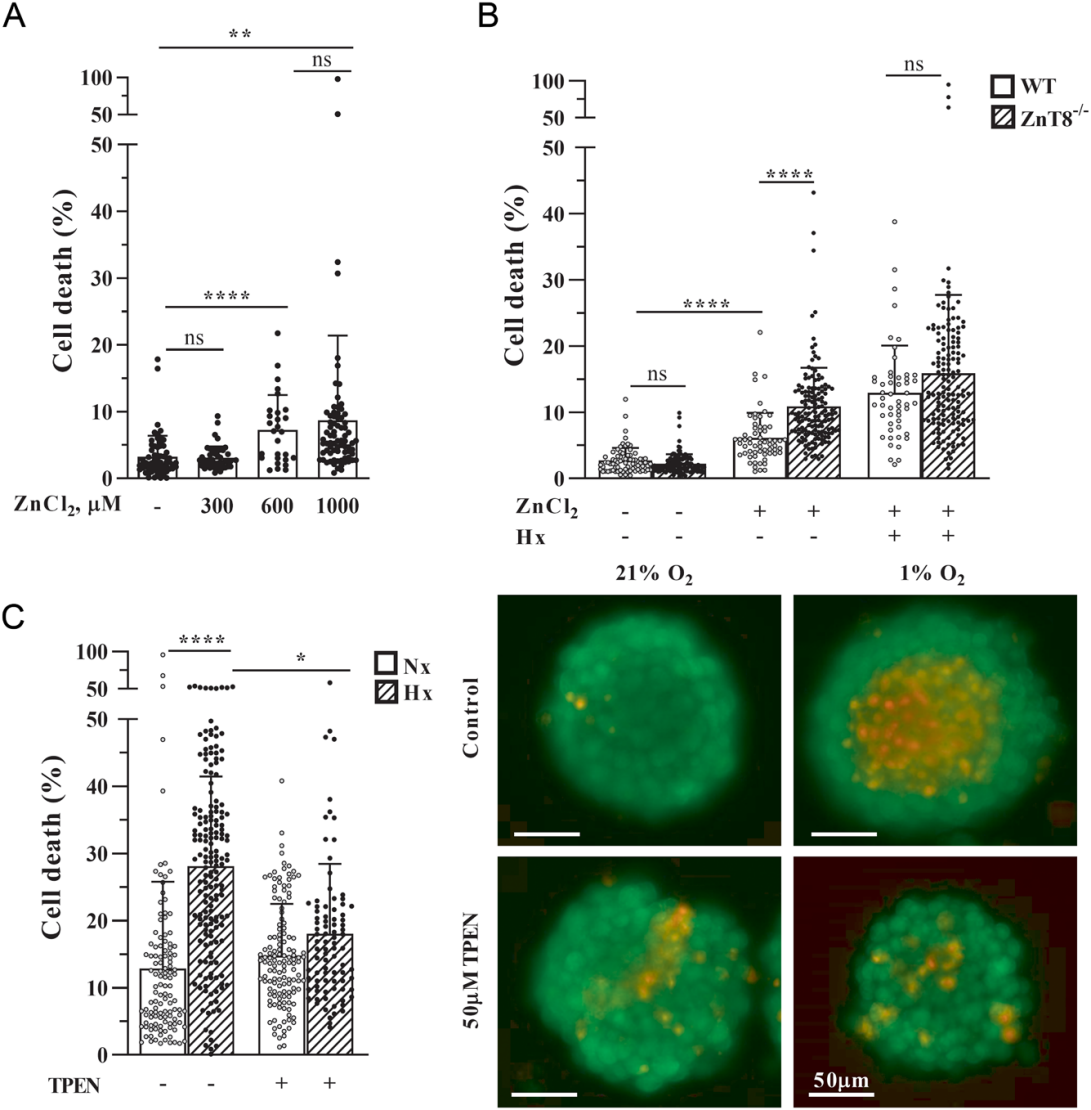
ZnT8-mediated changes of hypoxia-induced islet cell death rate depend on Zn^2+^. (A) Cell death in pancreatic islets from WT mice is induced by ZnCl_2_ in a dose-dependent manner. *n* (islet number) ≥ 30 per condition. (B) The proportion of dead cells was assessed in islets of WT and ZnT8^−/−^ mice exposed to 600 µM ZnCl_2_ in the presence of normoxia (21% O_2_, Nx) or hypoxia (1% O_2_, Hx) for 24 h. *n* (islet number) ≥ 50 per condition. (C) Islets of WT mice were exposed to normoxia or hypoxia for 24 h with or without 50 µM of the zinc chelator TPEN. *n* (islet number) ≥ 80 per condition. Representative microscopy images of pancreatic islets are depicted: staining of viable cells (calcein-AM, green) and cell death (PI, red). Data are expressed as mean ± s.d. ns, not significant; ^*^ *P* ≤ 0.05, ^**^P ≤ 0.01, ^****^P ≤ 0.0001. Generalized multivariate analysis was performed.

As shown in previous studies (Gerber *et al.* 2014), pancreatic islets deficient in zinc transporter ZnT8 exhibit reduced intracellular zinc levels. Thus, it was hypothesized that these lower intracellular zinc levels may protect pancreatic islets from cell death in situations of increased islet cell stress. Exposure of cells to increased zinc levels (which consecutively leads to higher intracellular (Vinkenborg *et al.* 2009) and in particular, cytosolic (Bellomo *et al.* 2011) zinc levels) increases the rate of cell death in pancreatic islets (Kim *et al.* 2000), which we could reproduce in the present study (Fig. 2A).

This effect was more pronounced in pancreatic islets lacking ZnT8. Furthermore, the protective effect of ZnT8 deficiency on hypoxia-induced cell death disappeared with exposure of islets to high zinc levels (Fig. 2B).

To simulate the effect of reduced ZnT8 activity on intracellular zinc levels, WT islets were treated with the zinc chelator TPEN, thus reducing intracellular zinc content (Vinkenborg *et al.* 2009). When exposed to hypoxic conditions, TPEN treatment was able to reduce the extent of cell death (Fig. 2C).

### ZnT8*/Slc30a8* knockout protects pancreatic islets from cytokine-mediated cell death in a zinc-dependent way

**Figure 3:**
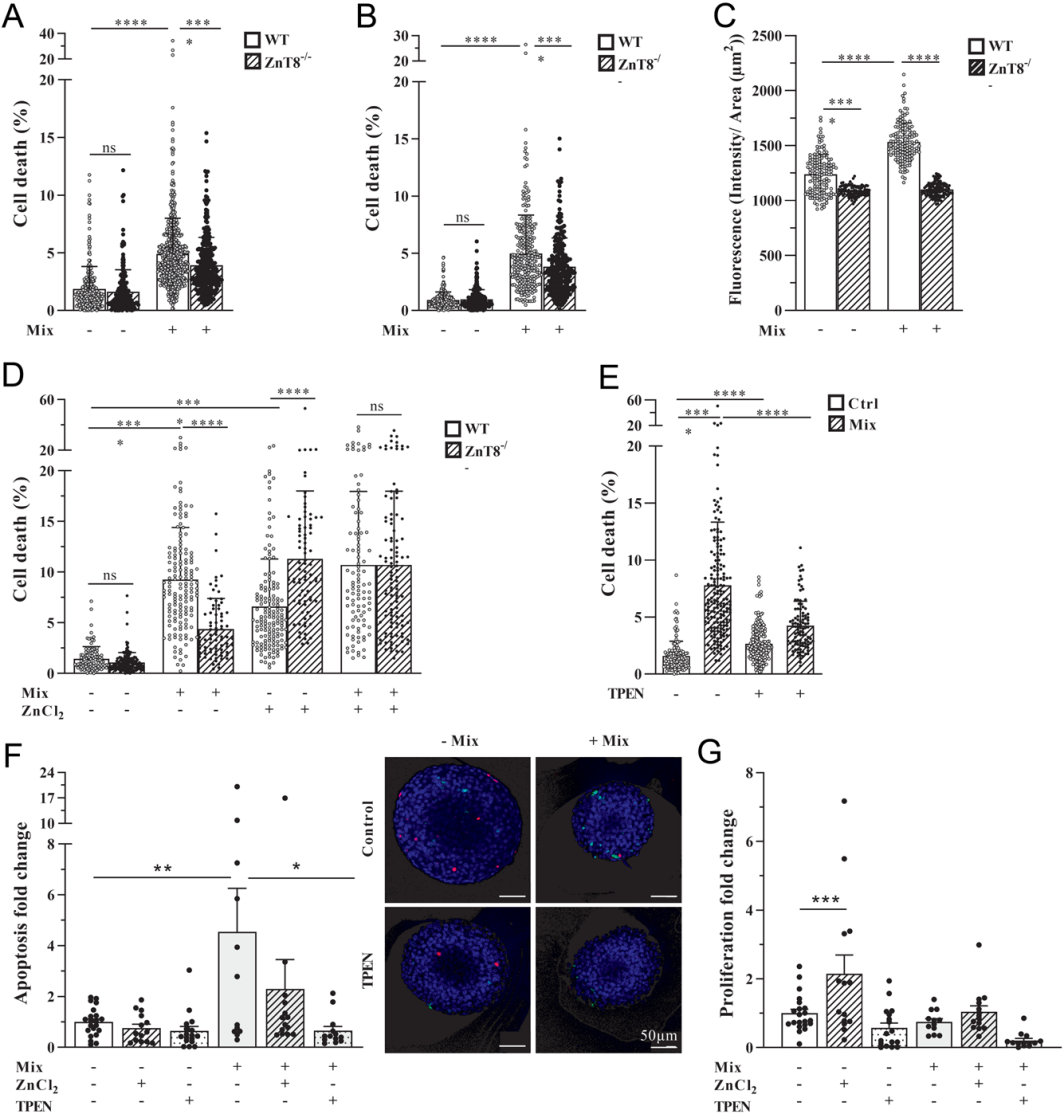
Cytokine-mediated cell death in pancreatic islets is dependent on the level of ZnT8 expression and Zn^2+^ concentrations. The proportion of dead cells in islets of WT and ZnT8^−/−^ mice at age of (A) ~14 weeks and (B) ~29 weeks is depicted after incubation of islets for 24 h with or without a mixture of cytokines (1000 U/mL TNF-α + 1000 U/mL IFN-γ + 50 U/mL IL1-β, Mix). *n* (islet number) ≥ 250 per condition. (C) The cytosolic zinc concentration was estimated applying FluoZin-3AM staining in islets of WT and ZnT8^−/−^ mice with and without a mixture of cytokines (Mix). Measurements were corrected for autofluorescence (no FluoZin condition). *n* (islet number) ≥ 80 per condition. (D) The proportion of dead cells in islets of WT and ZnT8^−/−^ mice is depicted after incubation of islets for 24 h with a mixture of cytokines (Mix), 600 µM ZnCl_2_ or both. *n* (islet number) ≥ 80 per condition. (E) The proportion of dead cells in WT islets is depicted after 24 h incubation with cytokine mixture (Mix) and/or the zinc chelator TPEN. *n* (islet number) ≥ 100 per condition. (F) Fold change of positively stained cells for apoptosis (TUNEL, vs control) and (G) for proliferation (PCNA, vs control) in WT pancreatic islets is depicted after incubation (24 h) with different combinations of cytokines (Mix), ZnCl_2_ or TPEN. Representative microscopy images of pancreatic islets are depicted: Staining of apoptosis (TUNEL, green), proliferation (PCNA, red) and DAPI (blue). *n* (islet number) ≥ 12 per condition. Data are expressed as mean ± s.d. ns, not significant; ^*^P ≤ 0.05, ^**^P ≤ 0.01, ^***^P ≤ 0.001, ^****^P ≤ 0.0001. Generalized multivariate analysis (A, B, D, E), Mann–Whitney *U* test (C) and ANOVA (F, G) were performed.

To further test the observed effects in another setting of increased cell death, we exposed pancreatic islets to a mixture of cytokines known to induce cell death. The combination of TNF-α, IFN-γ and IL-1β induced cell death in pancreatic islets from mice of different ages (Pierre Pirot 2008, Riboulet-Chavey *et al.* 2008). This effect was seen in both WT and ZnT8^−/−^ mice but was observed to a lesser extent in ZnT8^−/−^ islets (Fig. 3A and B).

The effect of *Slc30a8* knockout on intracellular zinc levels was explored using the fluorescent zinc probe FluoZin-3AM. This revealed decreased free zinc concentrations in ZnT8^−/−^ islets (as compared to WT islets) without and after exposure to the cytokine mixture (Fig. 3C).

Exposure to added zinc ions increased cell death in islet cells to a greater extent in ZnT8^−/−^ islets compared to WT islets, an effect that disappeared when islets were exposed to the cytokine mixture (Fig. 3D).

Treatment with TPEN was able to reduce cytokine-mediated islet cell death in WT islets (Fig. 3E).

To further assess the mechanisms by which zinc and the reduction/depletion of zinc influence cell death in WT islets, we assessed islet cell apoptosis by TUNEL staining as well as cell proliferation by PCNA staining. While changes in zinc concentrations by adding ZnCl_2_ or the zinc chelator TPEN did not change the proportion of apoptosis under control condition, cytokine-induced apoptosis could be reduced by the addition of TPEN (Fig. 3F).

Of interest, the proliferation rate was increased by zinc under control conditions (Fig. 3G).

### ZnT8 deficiency does not alter the response of islet insulin secretion to cytokine exposure

**Figure 4:**
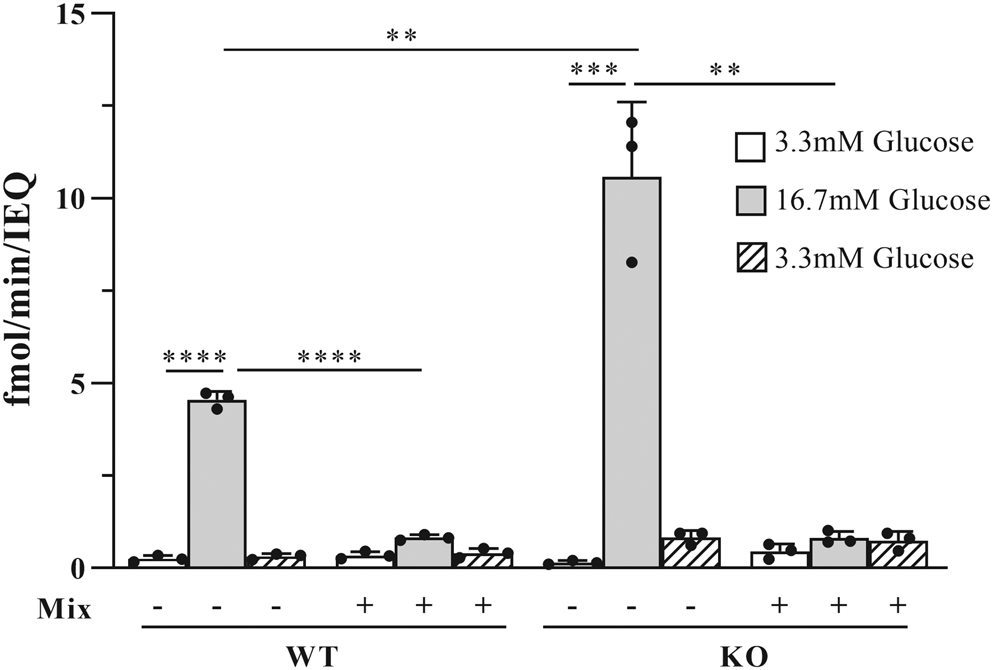
The effect of cytokine treatment on GSIS in pancreatic islets from ZnT8^−/−^ and WT mice. Mouse islets were incubated for 24 h with a cytokine mixture (1000 U/mL TNF-α + 1000 U/mL IFN-γ + 50 U/mL IL1-β, Mix). The insulin concentration (fmol/min/IEQ) in the supernatant was determined following incubation for 1 h in each 3.3 mM, 16.7 mM and again 3.3 mM glucose. Experiments were performed with three islet pools per condition (≥150 islets in each pool). Data are represented as mean ± s.d.^**^P ≤ 0.01, ^***^P ≤ 0.001, ^****^P ≤ 0.0001; Mann–Whitney *U* test was performed.

To test whether the response of glucose-stimulated insulin secretion to cytokine treatment is altered by a change in *Slc30a8* expression, we exposed islets of WT as well as ZnT8^−/−^ mice to low and high glucose levels by static incubation. As described previously (Nicolson *et al.* 2009), insulin secretion in ZnT8^−/−^ islets was higher after glucose stimulation compared to WT islets. However, after cytokine exposure, insulin secretion was reduced in both WT and ZnT8^−/−^ islets to a similar extent (Fig. 4).

### ZnT8 expression is reduced in pancreatic islets treated with cytokines

**Figure 5:**
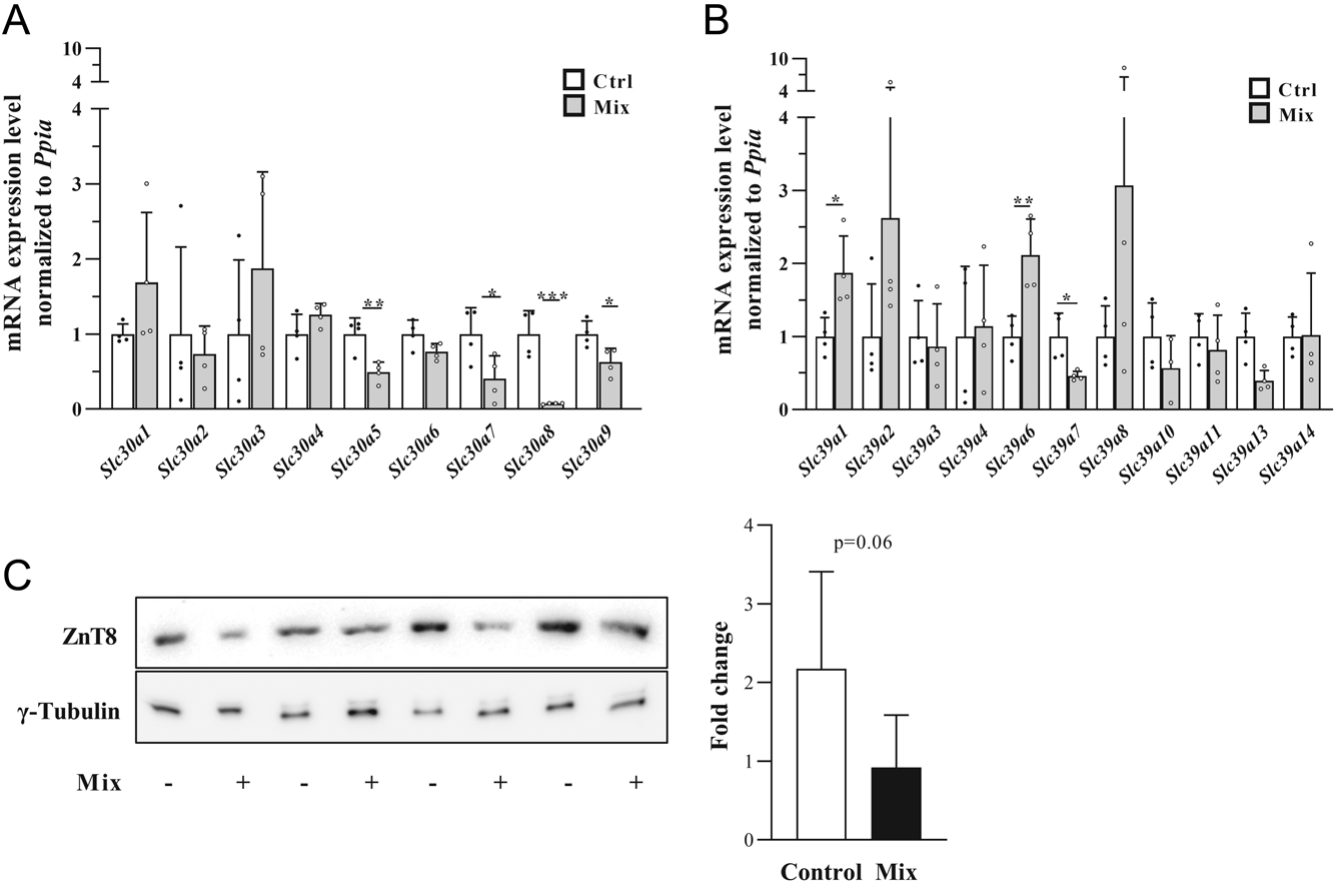
The effect of cytokine treatment on the expression of genes encoding zinc transporters in pancreatic islets. Mouse WT islets were incubated for 24 h with a cytokine mixture (Mix). (A) Slc30a (ZnT) mRNA expression, (B) Slc39a (ZiP) mRNA expression. (C) EndoC-βH5 cells were incubated for 24 h with a cytokine mixture (Mix) and protein expression was assessed by Western blot analysis. Data are expressed as mean ± s.d.*n* (replicates number) = 4; ^*^P≤ 0.05 ^**^P ≤ 0.01, ***P ≤ 0.001; Student’s *t*-test was performed.

The response of pancreatic islet cells to cytokine treatment was assessed by measurement of mRNA expression of zinc transport proteins (ZnT and ZIP families, Fig. 5A and B). This revealed downregulation of different zinc transporter mRNA, with the most pronounced effect on *Slc30a8.*Furthermore, ZnT8 also tended to be downregulated on protein level in a different system (human cell line), providing evidence of a very consistent effect across species.

## Discussion

This study provides further evidence that the regulation of intracellular zinc content is a fundamental process for islet cell function and survival. Elimination of the zinc transporter ZnT8 in pancreatic islet cells, which is accompanied by reduced intracellular zinc levels, results in less pancreatic islet cell death. This process can be modulated by changes in the extracellular zinc concentration to which islet cells are exposed, as demonstrated in the present study.

Early histochemical studies revealed a close connection between the zinc and insulin content of the pancreas and found that this (high) content depends on functional endocrine tissue (Scott & Fisher 1938). Later, it was shown that zinc is an essential component of insulin hexamers (Blundell *et al.* 1972) thought to be the fundamental form of insulin when stored in the insulin secretory granules of β-cells (Emdin *et al.* 1980). At the same time, it is well known that high intracellular concentrations of zinc are potentially cytotoxic. The cytotoxic effect of zinc ions can be modulated by chelation or competition for cell entry with other ions (Borovansky & Riley 1989), which was also demonstrated for pancreatic β-cells (Kim *et al.* 2000). Thus, the regulation of islet cell zinc content appears to be a fine balance between proper function and death of these cells.

Although intracellular zinc content in pancreatic islets, as well as their proper function, is impaired in mouse models lacking the zinc transporter ZnT8 (Nicolson *et al.* 2009), findings which were confirmed in the present study, knockout islets are more resistant to the cytotoxic effects caused by hypoxia and cytokine exposure. That intracellular zinc plays a central role in modulating hypoxia- and cytokine-induced damage is suggested by the fact that exposure to high zinc concentrations abolishes the observed cytoprotective effect. We assume that a high load of zinc ions and consequent increase in cytosolic zinc hamper the ability of ZnT8 to regulate intracellular zinc concentrations. In the absence of stressors such as hypoxia or cytokines, ZnT8^−/−^ islets are even more prone to zinc-induced cell death than WT islets, possibly because they are less armed with defensive mechanisms against high concentrations of intracellular zinc, for example, zinc-binding metallothioneins which have been shown to protect from Zn^2+^-induced cell death in different cell types (Wiseman *et al.* 2006, Smith *et al.* 2008). This corroborates our finding that hypoxia-induced expression of metallothioneins 1 and 2 (Mt1, Mt2) is impaired in pancreatic islets deficient in ZnT8 (Gerber *et al.* 2014).

Whereas high concentrations of zinc induce cell death in a wide range of cell types, it was also shown that zinc depletion negatively influences cell survival: in the rat insulinoma-derived β-cell line INS-1E, high zinc concentrations induced cell necrosis while zinc chelation induced apoptosis (Nygaard *et al.* 2014). However, in situations of cellular stress, these effects are probably shifted in favour of lower zinc concentrations, since the constitutively high concentrations of zinc in islet cells may exert an additive stress to the cell. From observations in neuronal tissues, where the interplay of hypoxic injury and zinc-mediated modulation of cell death has been studied extensively, it is known that zinc chelation by TPEN decreases apoptosis and cell death induced by oxygen and glucose deprivation (Liu *et al.* 2015, Wang *et al.* 2015, Zhang *et al.* 2017). This might also be true for pancreatic islet cells with a very high intracellular zinc content. In contrast, in the absence of exogenous stress, chelation of zinc might have adverse effects on islet cell survival as shown previously by Lefebvre *et al.* (2012) and also confirmed by our data demonstrating an increased cell death in islets treated with TPEN in the absence of hypoxia- or cytokine-mediated stress.

Additional analysis of cell apoptosis in our analysis revealed that in the absence of cytokine exposure, there was no significant influence of zinc on islet cell apoptosis. Thus, we assume that the observed increase in islet cell death by zinc is mainly exerted by non-apoptotic cell death. This is in accordance with previously published data showing increased necrosis, but not apoptosis, when zinc was added to pancreatic β-cells (Chang *et al.* 2003). However, zinc depletion was able to abrogate the effects of cytokines on apoptosis. Thus, regarding apoptosis, zinc is of limited effect in non-stressed β-cells even at higher concentration but becomes a major threat under conditions associated with increased apoptosis (i.e. cytokine exposure). The narrow line between pro-apoptotic and anti-apoptotic properties of zinc is well known from other settings, for example in cancer cells, where its effects vary widely depending on the cell type and other conditions (Franklin & Costello 2009).

While zinc depletion can be beneficial in situations of acute cell stress, it may compromise islet cell proliferation in the long run as suggested by our data and existing literature (Nygaard *et al.* 2014).

Of interest, both hypoxia (Gerber *et al.* 2014) and cytokine exposure (El Muayed *et al.* 2010) are known to mediate downregulation of the zinc transporter ZnT8 in pancreatic islets, which we confirmed in the present study, suggesting that this constitutes a protective mechanism, tasked with reducing intracellular zinc content and thus reducing the rate of cell death, an effect which can be abolished by adding excessive exogenous zinc. In this way, ZnT8 downregulation may counteract other cytokine-mediated mechanisms that are known to increase intracellular zinc content (e.g. increased expression of Zip8 (Besecker *et al.* 2008)) and which may be particularly harmful in pancreatic β-cells.

Other mechanisms contributing to an inflammatory stress protection by ZnT8 downregulation have been shown previously, namely by promoting an adaptive protective unfolded protein (UPR) response (Merriman & Fu 2019). However, this was shown in a tumour cell line. It remains to be determined whether such mechanisms also apply to primary islet cells.

In contrast to cell survival, our data showed that glucose-stimulated insulin secretion from isolated islets treated with cytokines was not altered in ZnT8^−/−^ islets compared to WT islets. Glucose-stimulated insulin secretion was however reduced in islets from both strains of mice when exposed to the cytokine mixture. Together with previous studies, this finding demonstrates consistent effects of cytokine exposure on insulin secretion over a broad range of cytokine concentrations (Kiely *et al.* 2007, Laporte *et al.* 2019). Among other factors, induction of oxidative stress and restriction of mitochondrial capacity for oxidising pyruvate contribute to this effect (Barlow *et al.* 2018).

As hypothesized by earlier studies, functional compensation by the *Slc30a7* isoform, which encodes ZnT7, may reduce the impact of *Slc30a8* deletion on islet function (Syring *et al.* 2016). However, this effect might be limited since we observed a reduced transcription of *Slc30a7* as well after cytokine treatment.

As a limitation of this study, using a global ZnT8 ko model, the effects observed in islets may not be fully attributed to pancreatic β-cells, because other islets cells (i.e. α-cells) express ZnT8. However, since most of the differences in the ratio of live to dead cells were observed in the centre of the rodent islets (which is populated almost exclusively by β-cells) and since the vast majority of pancreatic islet cells consists of β-cells, it can be assumed that β-cells are probably the most important contributor to the observed effects.

It remains to be defined to what extent a decreased rate of cell death (and thus a suggested preserved mass of insulin-secreting cells with proper function) contributes to a protective effect of ZnT8 loss-of-function in the context of T2DM. Recent studies suggest an increased insulin secretion capacity of β-cells with ZnT8 loss-of-function to be the reason for a lower risk to develop diabetes (Dwivedi *et al.* 2019). However, our data demonstrate that mice lacking ZnT8 exhibit slightly worsened long-term glucose control, despite the better resistance of islets to hypoxia- or cytokine-mediated cell death. It might be possible that certain compensation mechanisms are in place *in vivo* which cannot be stimulated *in vitro*, at least in mice. However, in line with our results, transgenic mice overexpressing the human *hZnT8 R325W* polymorphism (which is associated with a decreased susceptibility to T2DM) have reduced islet Zn^2+^ levels as well as higher glucose tolerance when fed a high-fat diet, as compared to their *hZnT8 WT* littermates (Li *et al.* 2017). In any case, future studies are likely to be necessary to understand the impacts of ZnT8 deletion as well as the action of presumed loss-of-function alleles (Kleiner *et al.* 2018) both in mice and in humans. An important goal is therefore to explain the apparent discordance between the impact of these alleles on β-cell function and survival vs glycaemic control and type 2 diabetes risk (Rutter & Chimienti 2015). Importantly, by demonstrating a mechanism through which ZnT8 inactivation may lead to resistance towards β-cell death in the longer term, while impairing glucose tolerance in the mouse in the short-term, our findings might provide an explanation for the protective effects towards diabetes of rare loss-of-function *SLC30A8* alleles in man (Dwivedi *et al.* 2019).

In conclusion, our study provides evidence for a decreased rate of cell death in islets lacking the zinc transporter ZnT8 as compared to WT islets when exposed to different cellular stressors, such as hypoxia and cytokines. Thus, targeting ZnT8 remains an appealing therapeutic strategy against diabetes.

## Supplementary Material

Supplementary table 1. Primers used for qPCR of mouse islets.

## Declaration of interest

G A R has received grant support from Les Laboratoires Serviers and from Sun Pharmaceuticals and is a consultant for Sun Pharmaceuticals.

## Funding

D J H was supported by MRC (MR/N00275X/1 and MR/S025618/1) and Diabetes UK http://dx.doi.org/10.13039/501100000361 (17/0005681) Project Grants. This project has received funding from the European Research Council http://dx.doi.org/10.13039/501100000781 (ERC) under the European Union’s Horizon 2020 research and innovation programme (Starting Grant 715884 to D J H). G A R was supported by a Wellcome Trust http://dx.doi.org/10.13039/100010269 Investigator Award (212625/Z/18/Z), MRC Programme grants (MR/R022259/1, MR/J0003042/1, MR/L020149/1) and by Diabetes UK http://dx.doi.org/10.13039/501100000361 (BDA/11/0004210, BDA/15/0005275, BDA 16/0005485) project grants. This project has received funding from the European Union’s Horizon 2020 research and innovation programme via the Innovative Medicines Initiative http://dx.doi.org/10.13039/501100010767 2 Joint Undertaking under grant agreement No 115881 (RHAPSODY) to G A R. This Joint Undertaking receives support from the European Union’s Horizon 2020 research and innovation programme and EFPIA. P A G received funding for this project from the Theiler-Haag and the Philhuman foundation.

## Author contribution statement

Maria Karsai: investigation, formal analysis, data curation, writing – original draft, visualization, project administration. Richard A Zuellig: investigation, methodology, writing – review and editing. Roger Lehmann: conceptualization, resources, writing – review and editing. Federica Cuozzo: investigation, formal analysis, writing – original draft. Daniela Nasteska: investigation, formal analysis, writing – original draft. Edlira Luca: investigation, formal analysis, writing – original draft. Constanze Hantel: investigation, formal analysis, writing – original draft. David J Hodson: investigation, methodology, validation, resources, writing – review and editing, funding acquisition. Giatgen A Spinas: conceptualization, writing – review and editing. Guy A Rutter: conceptualization, methodology, resources, writing – review and editing, funding acquisition. Philipp A Gerber: conceptualization, formal analysis, resources, data curation, writing – original draft, visualization, supervision, funding acquisition.
