# Supplementary material for: Lack of ZnT8 protects pancreatic islets from hypoxia- and cytokine-induced cell death
Source: J Endocrinol. 2022 Jan 11;253(1):1–11. doi: 10.1530/JOE-21-0271 (PMC8859919; doi:10.1530/JOE-21-0271)
Supplement: Supplementary table 1. Primers used for qPCR of mouse islets. [file supplementary_table_1.pdf]

Supplementary table 1. Primers used for qPCR of mouse islets.

Gene	Sequence 5' to 3'
Cyclophilin A fwd	TATCTGCACTGCCAAGACTGTA
Cyclophilin A rev	CCACAATGCTCATGCCTTCTTTCA
Slc30a1 fwd	ACTCAGTGTGCCCTGAAGCA
Slc30a1 rev	GCATCCTTCCCAGAATGCA
Slc30a2 fwd	CCCGACCAGCCACCAA
Slc30a2 rev	CCAAGGATCTCGGCTCGAT
Slc30a3 fwd	GGTGGTTGGTGGGTATTTAGCA
Slc30a3 rev	CAAGTGGGCGGCATCAGT
Slc30a4 fwd	CATCGCTGCCGTCCTCTAC
Slc30a4 rev	ATTTGCCATGTATCCACCTACAAG
Slc30a5 fwd	CTGTTTGCTGCCCTGATGAG
Slc30a5 rev	CGGCCATACCCATAGGAGAA
Slc30a6 fwd	TGAGCCCGGTTATTCTTCTGA
Slc30a6 rev	CCGCGATTAAGACCCAAACTAT
Slc30a7 fwd	ATGTTGCCCCTGTCCATCAAGG
Slc30a7 rev	TCGGAGATCAAGCCTAGGCAGT
Slc30a8 fwd	CGCAGTTGATGGCGTGATC
Slc30a8 rev	TGGTTCACAGTCAGTGACCAGAT
Slc30a9 fwd	GTAGGGACATTGGCTGCTTAGG
Slc30a9 rev	TTCAACACAGGCTGGCTGAA
Slc30a10 fwd	TTTCCCCTCCCTGGATCAG
Slc30a10 rev	ACACAGGCTCAAAGAAACGAGAT
Slc30a1 fwd	ACTCAGTGTGCCCTGAAGCA
Slc30a1 rev	GCATCCTTCCCAGAATGCA
Slc30a2 fwd	CCCGACCAGCCACCAA
Slc30a2 rev	CCAAGGATCTCGGCTCGAT

Slc30a3 fwd	GGTGGTTGGTGGGTATTTAGCA
Slc30a3 rev	CAAGTGGGCGGCATCAGT
Slc30a4 fwd	CATCGCTGCCGTCCTCTAC
Slc30a4 rev	ATTTGCCATGTATCCACCTACAAG
Slc30a5 fwd	CTGTTTGCTGCCCTGATGAG
Slc30a5 rev	CGGCCATACCCATAGGAGAA
Slc30a6 fwd	TGAGCCCGGTTATTCTTCTGA
Slc30a6 rev	CCGCGATTAAGACCCAAACTAT
Slc30a7 fwd	ATGTTGCCCCTGTCCATCAAGG
Slc30a7 rev	TCGGAGATCAAGCCTAGGCAGT
Slc30a8 fwd	CGCAGTTGATGGCGTGATC
Slc30a8 rev	TGGTTCACAGTCAGTGACCAGAT
Slc30a9 fwd	GTAGGGACATTGGCTGCTTAGG
Slc30a9 rev	TTCAACACAGGCTGGCTGAA
Slc30a10 fwd	TTTCCCCTCCCTGGATCAG
Slc30a10 rev	ACACAGGCTCAAAGAAACGAGAT
Slc39a1 fwd	CAGACGTGGTCAGGGACATTAG
Slc39a1 rev	TGTACTGCCTGGCCTCTGACT
Slc39a2 fwd	TGGCGGCCACCATACAG
Slc39a2 rev	AACACCACAAGCCCCTTATG
Slc39a3 fwd	TGTCAGCTTCTCCTATGGCTTGT
Slc39a3 rev	GGATCCCGCCTGCACTAATA
Slc39a4 fwd	GGGCCGTGTGAAAAGTGTCT
Slc39a4 rev	GGCTTGTCAGGTTTGCCTAGA
Slc39a5 fwd	CGAGCTGCCCCACGAA
Slc39a5 rev	AAAGGATAACCCTTCCTGAAGCA
Slc39a6 fwd	ACATTGGCCTGGATGGTGAT
Slc39a6 rev	AAGGCCGTCACTGAAATTGTG
Slc39a7 fwd	GCTGGGTCCTGCCATTCA

Slc39a7 rev	CAGCACAGACACTGTTGCTACGT
Slc39a8 fwd	GTACGCAGGAGACATCGAATTG
Slc39a8 rev	TGCCTTCCCGCGTTGA
Slc39a9 fwd	GGACCAGGCTGGCTTCAAA
Slc39a9 rev	CCAGCTCTAAGGAGGCAGAAAC
Slc39a10 fwd	GCCCTTCACCAGAGACCAATAA
Slc39a10 rev	CCTCCTGACCTTCACTGACTTCA
Slc39a11 fwd	CTCACCTGGGTGCTACAGAAGAC
Slc39a11 rev	CAATGCAGGGTCCAAGTTCA
Slc39a12 fwd	GGTTGTAAATTTGTCCTGCATGAA
Slc39a12 rev	TTGGGCTTGGGTTGTGTTG
Slc39a13 fwd	AGGAATGTCAACTGGAAGAATGC
Slc39a13 rev	GGTGTCAGCCAAGGCAAATAGT
Slc39a14 fwd	GAGTGGGCCGGGATAATGTT
Slc39a14 rev	AAAGCACGTGGAGAGGTTCCT
